# Non-invasive methods characterise the world’s largest tiger shark aggregation in Fuvahmulah, Maldives

**DOI:** 10.1038/s41598-024-73079-3

**Published:** 2024-09-23

**Authors:** Lennart Vossgaetter, Tim Dudeck, Jamie Crouch, Maiah Cope, Tatiana Ivanova, Ibrahim Siyan, Abdullah Niyaz, Mohamed Riyaz, Gonzalo Araujo

**Affiliations:** 1 Leibniz Centre for Tropical Marine Research, 28334 Bremen, Germany; 2 University of Bremen, 28334 Bremen, Germany; 3 Fuvahmulah Dive School, Fuvahmulah, 18011 Maldives; 4 Marine Research and Conservation Foundation, Lydeard St Lawrence, Somerset, UK; 5 Environmental Science Program, Department of Biological and Environmental Sciences, College of Arts and Sciences, Qatar University, Doha, Qatar

**Keywords:** Marine megafauna, Site fidelity, Gestation, Photo ID, LIR, Maximum-likelihood models, Population dynamics, Tropical ecology, Ecology, Biooceanography, Ichthyology

## Abstract

Tiger sharks are apex predators with a circumglobal tropical and warm-temperate distribution, with a general lack of population data for the central Indian Ocean. In Fuvahmulah, Maldives, tiger sharks display frequent use of the harbour area, attracted by discarded fish waste. Here, we document the population structure, residency, and reproductive characteristics of the world’s largest known tiger shark aggregation in a geographically-restricted area. Using non-invasive methods, photo identification and laser photogrammetry, we identified 239 individual tiger sharks over a 7-year study period. The aggregation was female-dominated (84.5%), with both large juveniles and adults present. Adult females were resighted over the entire study period displaying strong inter- and intra-annual site fidelity. Modelled residency using maximum likelihood methods suggests they spent 60.7 ± S.E. 7.5 days in Fuvahmulah, with a larger aggregation size, shorter residence periods and longer absence periods compared to juvenile females. Prolonged abdominal distensions of adult females indicate they likely stay near Fuvahmulah during gestation and reproduce biennially. Fuvahmulah seems to provide suitable conditions for gestation given the year-round provision of food and warm waters, exhibited by strong site fidelity and temporal residency. Our results show indications of a thriving population within the confines of protected waters.

**Supplementary Information:**

The online version contains supplementary material available at 10.1038/s41598-024-73079-3.

## Introduction

Across the globe, iconic predators such as sharks, wolves and lions are disappearing at the hands of human development. Most of the remaining predators now exist in only a fraction of their historical range^1,2^. In the ocean, this decline is especially evident in pelagic sharks and rays with many populations declining by > 70% since 1970 ^3^. More than one third of all elasmobranch species are now threatened with extinction^4^. Yet predators, such as sharks, are essential for ecological balance due to their top-down regulatory impact on food webs^5–7^. Sharks not only support ecosystem stability but can also provide economic value through tourism, benefiting dive operators, local tourism industries and governmental bodies^8–10^. While live sharks offer both economic and ecological value, dead sharks play an economic role for fisheries and local livelihoods worldwide^11^.

**Fig. 1:**
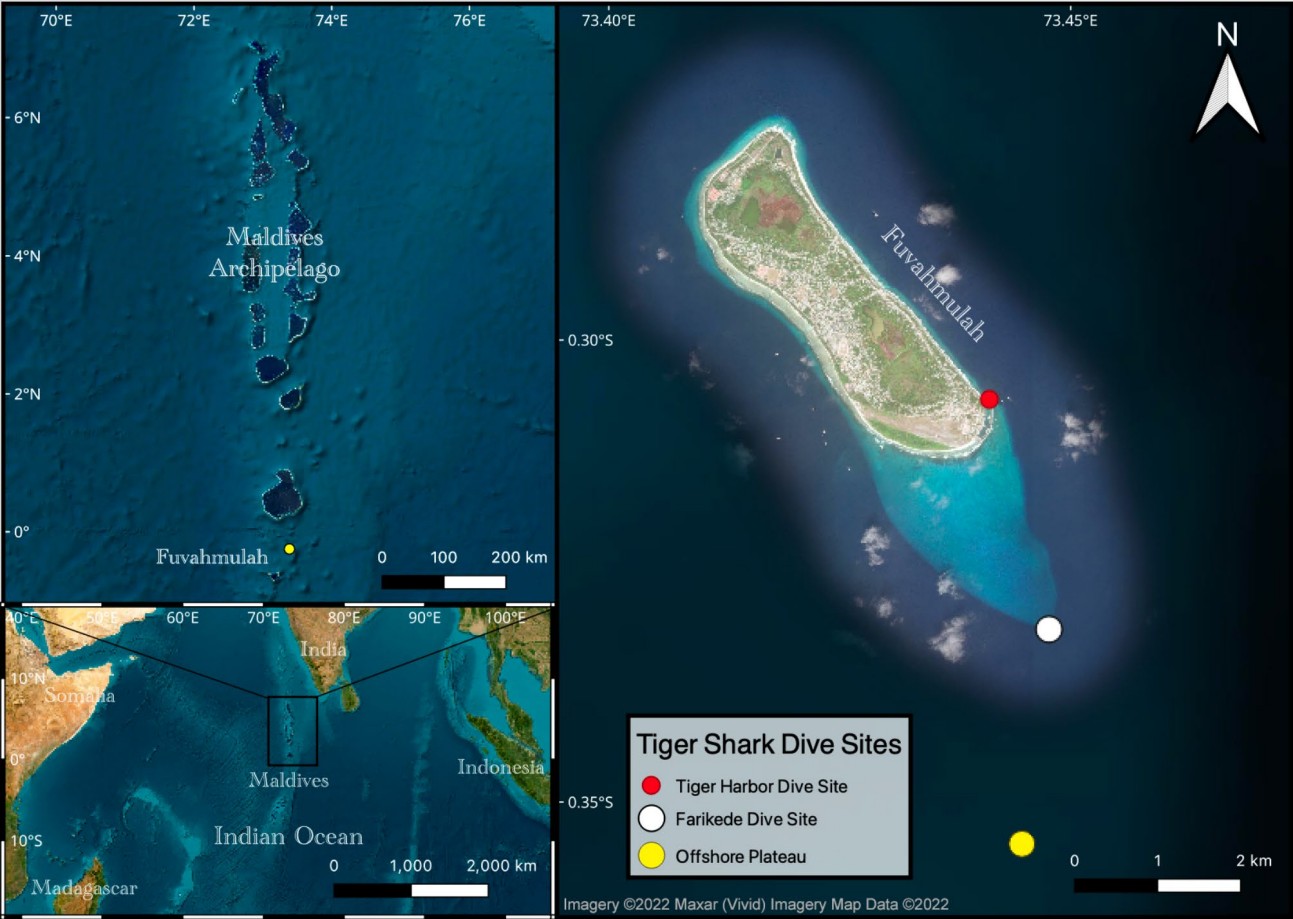
Location of Fuvahmulah within the Maldives Archipelago in the Indian Ocean. The dive sites, where tiger sharks are frequently spotted and where most footage originates from, are marked in the map (Map was created using QGIS 3.28.1-Firenze, URL: https://qgis.org/download/).

In the Maldives, fishing for sharks was a common practice and resulted in a decrease of reef shark populations at dive sites in the 1990s^12,13^. As a consequence, dive tourism declined leading to significant economic losses for local dive operators^13^. In response, the Maldivian government introduced legislation to protect sharks in their entire exclusive economic zone in 2010 creating one of the largest shark sanctuaries in the world with an area of 916,011 km^2^^  14,15^. The Maldives is home to a large diversity of elasmobranchs^8^ but lacks scientific information about critical habitats, behaviour and ecology with the exception of whale sharks *Rhincodon typus* and manta rays, *Mobula alfredi* and *Mobula birostris* (e.g. ^16,17^). Recently, a large aggregation of tiger sharks *Galeocerdo cuvier* was reported surrounding the oceanic island of Fuvahmulah in Southern Maldives (see Fig. 1). Previously neglected as a tourism destination, thousands of people now travel to Fuvahmulah annually to dive with this iconic species (Fuvahmulah dive centres, pers. comm.). The Island is a designated UNESCO Biosphere Reserve but scientific data on any of the local fish populations is lacking due to the recency of its commercial attention^18^.

Tiger sharks are large apex predators with a circumglobal distribution in warm-temperate and tropical waters^19^. In the Indian Ocean, females reach maturity at a total length (TL) of 3.0–3.5 m, whereas males mature at 2.8–3.2 m^20–22^, with a maximum TL of 5.5 m^23^. The tiger shark is the only member of the family Carcharhinidae with aplacental viviparous (ovoviviparous) reproduction^24^. Their adaptability as generalist predators allows them to function as apex and meso predators in different ecosystems^25^. The species is globally assessed as Near Threatened in the IUCN Red List of Threatened Species with decreasing population trends^19^. Due to the tiger shark’s low reproductive output and genetic diversity, the species is vulnerable to overexploitation by targeted shark fisheries and shark control programs, and as bycatch in commercial and artisanal fisheries globally^19,26^. The reproductive cycle is currently one of the most enigmatic aspects of their biology^25^, even though highly relevant to species management^27^. Initial work from the North Atlantic suggests tiger sharks follow a biennial cycle^28,29^. Castro (2009) proposed that they have a gestation period of 12 months with a synchronous reproductive cycle, where mating occurs before pregnant females pup in late spring and summer^24^. In Hawaiian tiger sharks however, a triennial cycle was observed with a gestation period of 15–16 months^30^. Studies using nuclear markers (microsatellites) revealed two major populations, one in the Atlantic and one in the Indo-Pacific^31,32^. Due to the genetic connectivity within the Indo-Pacific, several studies assumed that Indo-Pacific tiger sharks follow a triennial reproductive cycle e.g.^22,33^. However, Manuzzi et al. (2022) used genomic analysis to highlight the occurrence of localised cryptic populations of tiger sharks in Eastern Australia at finer geographical scales than previously understood^34^. As different populations may have differing life history traits, reproductive cycle generalisations should be regarded with caution^35^. With a lack of data throughout the rest of the Indo-Pacific and the possibility of localised populations, the reproductive cycle length remains unclear, particularly for tiger sharks in the Maldives.

In the Indo-Pacific, the species’ space use has been extensively studied in Hawai’i^36,37^, Eastern Australia^33,38^, Western Australia^39,40^, Southeast Africa^41,42^ and the Eastern Pacific^43,44^. Despite tiger sharks having been studied in the Indian Ocean, movements to–from the Maldives have not yet been identified^26,41,45^. Their space use is highly variable depending on the location, habitat and life stage with large intraspecific variation. Tiger sharks can migrate vast distances (i.e. 1000s of km), but have also been shown to display site fidelity in adults and residency in juveniles^33,42,43,46^. In Hawai’i, the species has been shown to display partial migrations in which some individuals display residency, whereas others, usually adult females, migrate offshore^37^. In the Galapagos Marine Reserve (GMR), Ecuador, juvenile and subadult tiger sharks remain resident year-round with tracked individuals spending a remarkable 93% of their time within the GMR^43^. In the Bahamas, female subadult and adult tiger sharks display site fidelity to a large, shallow sand bank for reproductive purposes during the boreal winter. The warm and protected waters are used for gestation^47^. In Eastern South Africa and Mozambique, adult tiger sharks exhibited relatively restricted space use along the continental shelf likely linked to abundant food sources in the region^41^.

In Fuvahmulah, the local traditional tuna fishery dates back for centuries and discards have historically been tossed into the ocean^48^. According to local anecdotal information, large sharks have always gathered around fishing boats waiting for fish discards and depredation opportunities (M. Ibrahim, pers. comm.). After the harbour was built in 2004, fish waste started accumulating in the surrounding harbour area, which since 2017 serves as a dive site for tiger shark tourism. The first registered local dive school in Fuvahmulah, Fuvahmulah Dive School (FDS), began collecting videos and photos of tiger sharks in 2016. The footage allows for the recognition of individuals through external markings and pigmentation patterns over time^49^. Photographic identification (photo-ID) is a wide-spread, non-invasive method to study populations of megafauna^50^. The method has been applied to a variety of species with distinct markings such as white sharks (e.g.^51^), striped hyenas (e.g.^52^), whale sharks (e.g.^53^), manta rays (e.g.^54^), cetaceans (e.g.^55^), marine turtles (e.g.^56^), anurans (e.g.^57^) and others, to describe population structure, abundance, residency, demographics, and animal movement between study sites. The fundamental assumption of photo-ID is that the natural markings are unique enough to reliably distinguish individuals within a population and that these do not change over time^50,58^. Most research exploring the demographic structure, reproductive patterns and residency behaviour of tiger sharks has relied on either fisheries-dependent data (e.g.^59^) or acoustic and satellite telemetry (e.g.^60^). Through the recent commercial development of tiger shark diving in Fuvahmulah, a substantial quantity of footage has been collected by dive centres and guests. With increased camera usage by recreational divers, citizen science can aid significantly in the data collection for photo-ID studies^61–63^. While previous research has employed photo-ID to study tiger sharks^64,65^ applying up to 14 visual traits for identification^49^, this study represents the first extensive investigation of the species with this method.

Due to limited scientific information on tiger sharks in the Indian Ocean, there exists an imminent need to determine reproductive parameters, population structure and critical habitats. In the present study, we use photographic data from 2016 to 2023 and laser-photogrammetry to investigate: (a) the demographic structure of tiger sharks visiting Fuvahmulah, (b) reproductive indications and (c) the level of site fidelity/residency to this oceanic island in the Maldives (Fig. 1). Hereby, this study presents the first scientific description of this tiger shark aggregation, with implications for the management and conservation of tiger sharks in the Maldives.

## Results

### Survey effort

Between Dec 7th 2016 and Sep 30th 2023, we collected footage from a total of 788 separate dive surveys: 772 at the Tiger Harbor (TH) dive site, six at Farikede (FK) and ten from Offshore Plateau (OP) (Fig. 1). We saved 32,495 photographs and frame grabs from video material of sufficient quality to identify individual sharks.

### Photo identification

**Fig. 2:**
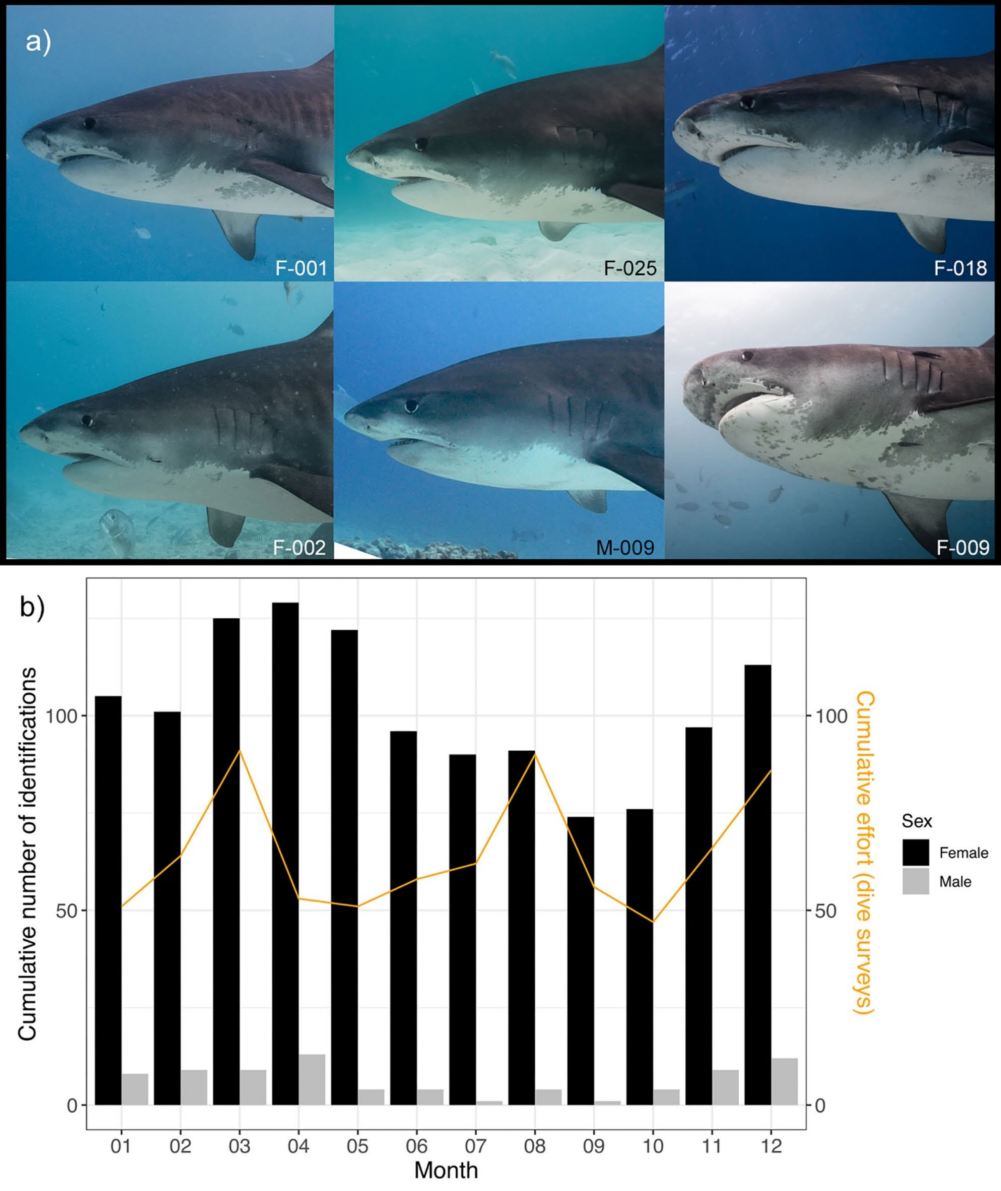
(**a**) Countershading delineation in six different tiger sharks. The six different individuals (F-001, F-002, F-018, F-009, F-011, F-025) display intraspecific variation of the countershading delineation anterior to the pectoral fins. This feature was most useful in differentiating the individuals. (**b**) Cumulative number of identifications of tiger sharks by sex per month visiting tiger harbour. The orange line indicates the cumulative number of dive surveys per month.

A total of 239 individual tiger sharks were identified, exhibiting a significant female bias (F = 202, M = 37, Chi-squared test, *χ*^*2*^ = 113.9, *p* < 0.0001). We logged 6,035 individual encounters throughout the study period (encounter meaning one identification of one individual). The majority (*n* = 5,986, 99.3%) of these encounters took place in TH. Of all encounters, 93.7% (*n* = 5,653) were females. Most individuals (*n* = 186) were sighted on more than one dive survey resulting in a resighting rate of 77.8%. On average, individuals were resighted on 25.3 (SD = 28.8) dive surveys with a maximum of 128 encounters for individual F-011, being present on 16.6% of all dive surveys at TH. A total of 89 sharks (37.2%) were seen in only one year, whereas 150 individuals (62.8%) were sighted over multiple years (≥ 2) and 53 individuals (22.2%) were seen ≥ 5 years (Supplementary Fig. S1). On average, we encountered 10.4 (SD = 5.6) individuals per dive survey, with a maximum of 40 sharks encountered during a single dive survey of 61 min length on Apr 21st 2022. During a one-year period of highest sampling effort from July 2021 until June 2022, we identified 186 individuals. When inspecting cumulative identifications per month, males tend to be almost absent from TH during the months of July (*n* = 1) until September (*n* = 1) and had highest sighting rates from November until April. Although females were present year-round, female sighting rates were significantly higher during the months of the Northeast monsoon including transitional months compared to the months of Southwest monsoon (Fig. 2b, Student’s t-test, *t*=-4.778, *p* = 0.0007). The number of dive surveys did not play a significant role in determining the number of identifications (Fig. 2b).

### Size estimates

Total length (TL) was visually estimated for a total of 213 sharks: 175 (82.16%) females and 38 males. From those estimated, we used laser photogrammetry to measure 52 individuals on 65 occasions from November 2021 until April 2022 at TH. There was no significant difference between estimates and measured values (*t*=−0.7745, *p* = 0.4422) with a mean difference of -2.1 cm ranging from − 49.0 cm to 34.1 cm (see Supplementary Table S1). Therefore, visual size estimates were assumed to be sufficient for approximate TL estimation of the tiger sharks.

**Fig. 3:**
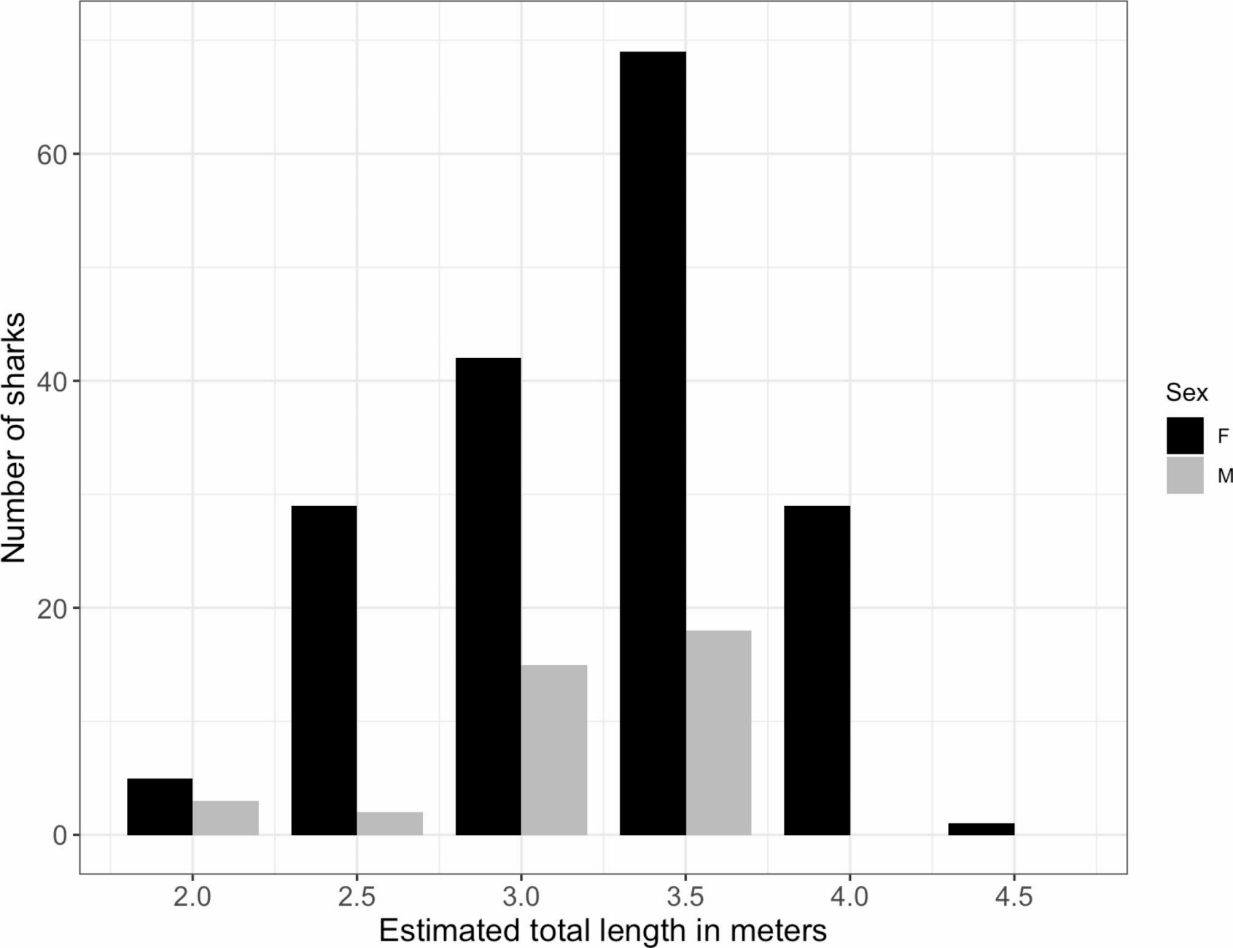
Total length (TL) of visual size estimates from 213 sharks incremented by 0.5 m. All females 3.5 m and larger were considered adults. All males 3.0 m and larger were adults.

Tiger sharks ranged from 2.0 to 4.5 m in TL with a mean size of 3.24 m (*n* = 213, SD = 0.52 m). Males ranged from 2.0 m to 3.5 m in size, whereas females ranged from 2.0 to 4.5 m. There was no significant difference between the mean size of males and females (*t* = 1.5541, *p* = 0.1253). However, all sharks that were estimated > 3.5 m TL were females. Most sharks were larger than the size at maturity resulting in 57.0% of the females considered adults, which were responsible for 68.5% (*n* = 4136) of all encounters (Fig. 3). Male tiger sharks encountered in TH all ranged from 3.0 to 3.5 m and were sexually mature. Males ranging from 2.0 to 2.5 m were only encountered at OP (*n* = 5). Female tiger sharks of all size classes were encountered at TH suggesting the presence of juvenile and adult individuals.

### Reproductive indications

**Fig. 4:**
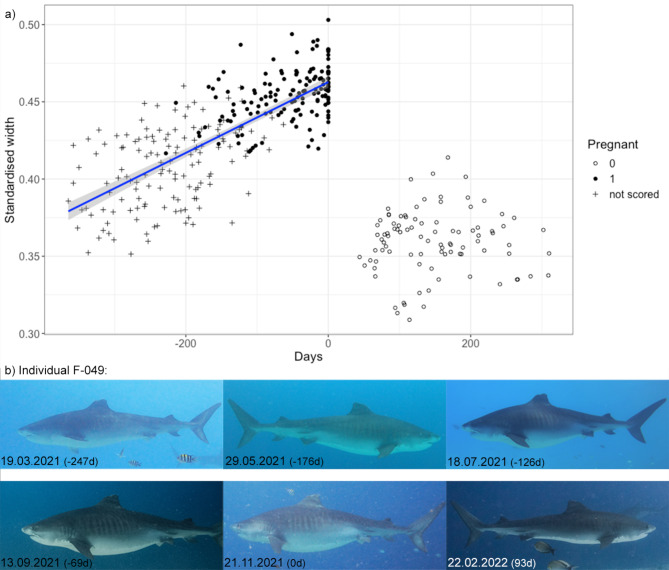
(**a**) Standardised width of presumably pregnant sharks over time. Day 0 indicates the last measurement before the sharks’ absence period, where we presume parturition may take place. Sharks scored 1 or 0 were visually assessed as ‘pregnant’ or ‘not pregnant’, while ‘not scored’ refers to a shark’s appearance, where we did not infer pregnancies based on their visual appearance (see Methods for more details). A linear regression model, including standard error, is fitted to the data until Day 0. (**b**) An example of one individual’s presumed pregnancy (F-049). Number in brackets provides a corresponding day value to a). The standardised width of this shark increased until day 0 followed by a period of absence for 93d. Upon its return, the standardised width of this shark had significantly declined.

A total of 54 pregnancies of 39 individuals (33.9% of all adult females) were documented from tiger sharks present in Fuvahmulah. For 35 pregnancies of 32 sharks, picture quality was sufficient to assess their standardised width over time. There was a significant difference in standardised widths between sharks considered pregnant [median(IQR) = 0.45 (0.44–0.46)] and sharks that consequently returned [median(IQR) = 0.36(0.35–0.37)] after an absence period with presumed parturition (*U* = 0, *p* < 0.0001). Figure 4a depicts the increase of the standardised width over time until they were not sighted at the dive site anymore. When they return, a significant change in their body width was obvious, highlighting the morphological difference after differing periods of absence (Fig. 4b). The timing, size and body development suggests these female tiger sharks return from parturition at a different location. During continuous sampling effort, 40 pregnancies of 36 individuals were recorded, allowing quantification of their absence periods. The median sharks’ absence for presumed parturition was 97 days ranging from 35 to 345 days (IQR = 69–126 d).

Two consecutive pregnancies were recorded for nine individuals and three consecutive pregnancies were recorded for three individuals. Consecutive periods of gestation and parturition were not significantly different from an expected two-year period (Chi-squared test, *χ*^*2*^ = 2.7783, *p* = 0.9994). Footage of pregnant sharks was separated on average by 788.7 (SD = 69.0) days or 2.16 years.

### Residency and lagged identification rate

The residency models were run for (i) the entire population, (ii) adult females, and (iii) juvenile females. Model H, which included parameters for emigration, reimmigration and mortality of tiger sharks, had the lowest QAIC indicating the best fit for the data of all three data sets (Table 1). According to the model, overall tiger sharks spent a mean of 65.5 ± S.E. 76.1 [95%CI (55.5–79.4)] days in Fuvahmulah and a mean of 107.9 ± S.E. 14.7 [95%CI (82.2-139.7)] days away. Aggregation size was estimated to be 43.1 ± S.E. 3.3 [95%CI (37.2–50.4)] individuals present in the study area on any given day.

**Table 1:** Residency model parameters as preset in SOCPROG 2.9 ^101^ and goodness of fit assessed through the Quasi-akaike Information Criterion (QAIC).

Model	Model description	Parameters	(i)∆ QAIC	(ii)∆ QAIC	(iii)∆ QAIC
A	Closed	1/a1 = *N*	6129.0	4284.9	2791.3
B	Closed	a1 = *N*	6129.0	4284.9	2791.3
C	Emigration/mortality	a1 = Emigration rate; 1/a2 = *N*	3695.2	3180.8	530.0
D	Closed: Emigration + reimmigration	a1 = Emigration rate; a2/(a2 + a3) = proportion of population in study area at any time	170.6	6.6	537.5
E	Emigration/mortality	a1 = *N*; a2 = Mean residence	3695.2	3180.8	530.0
F	Emigration + reimmigration + mortality	*NA*	3218.1	2997.7	490.9
G	Emigration + reimmigration	a1 = *N*; a2 = Residency time in; a3 = residency time out	170.6	6.6	537.5
H	Emigration + mortality + reimmigration	a1 = *N*; a2 = Residency time in; a3 = Residency time out; a4 = Mortality	0.0	0.0	0.0

(i) shows model results for the entire population, (ii) for adult females, and (iii) for juvenile females. N is the population size.

Similarly, adult female tiger sharks spent a mean of 60.7 ± S.E. 7.5 [95% CI (50.2–72.9)] days in Fuvahmulah and a mean of 110.4 ± S.E. 15.8 [95% CI (81.2–135.5)] days away. Aggregation size was estimated to be 25.9 ± S.E. 2.2 [95% CI (22.0–30.5)] individuals present in the study area on any given day. In contrast, juvenile female tiger sharks spent a mean of 93.0 ± S.E. 42.7 [95% CI (32.7–182.1)] days in Fuvahmulah and a mean of 76.9 ± S.E. 55.7 [95% CI (19.3–219.7)] days away, whereas the aggregation size was estimated to be 16.0 ± S.E. 2.1 [95% CI (12.0–20.2)] individuals present in the study area on any given day. According to the model results, juvenile females spent shorter time periods away from the study site than adults, while remaining for longer periods when present. Their overall aggregation size was also considerably lower, which is consistent with the amount of juveniles vs. adult females identified. The results for all tiger sharks was similar to the results for the adult females except for aggregation size.

**Fig. 5:**
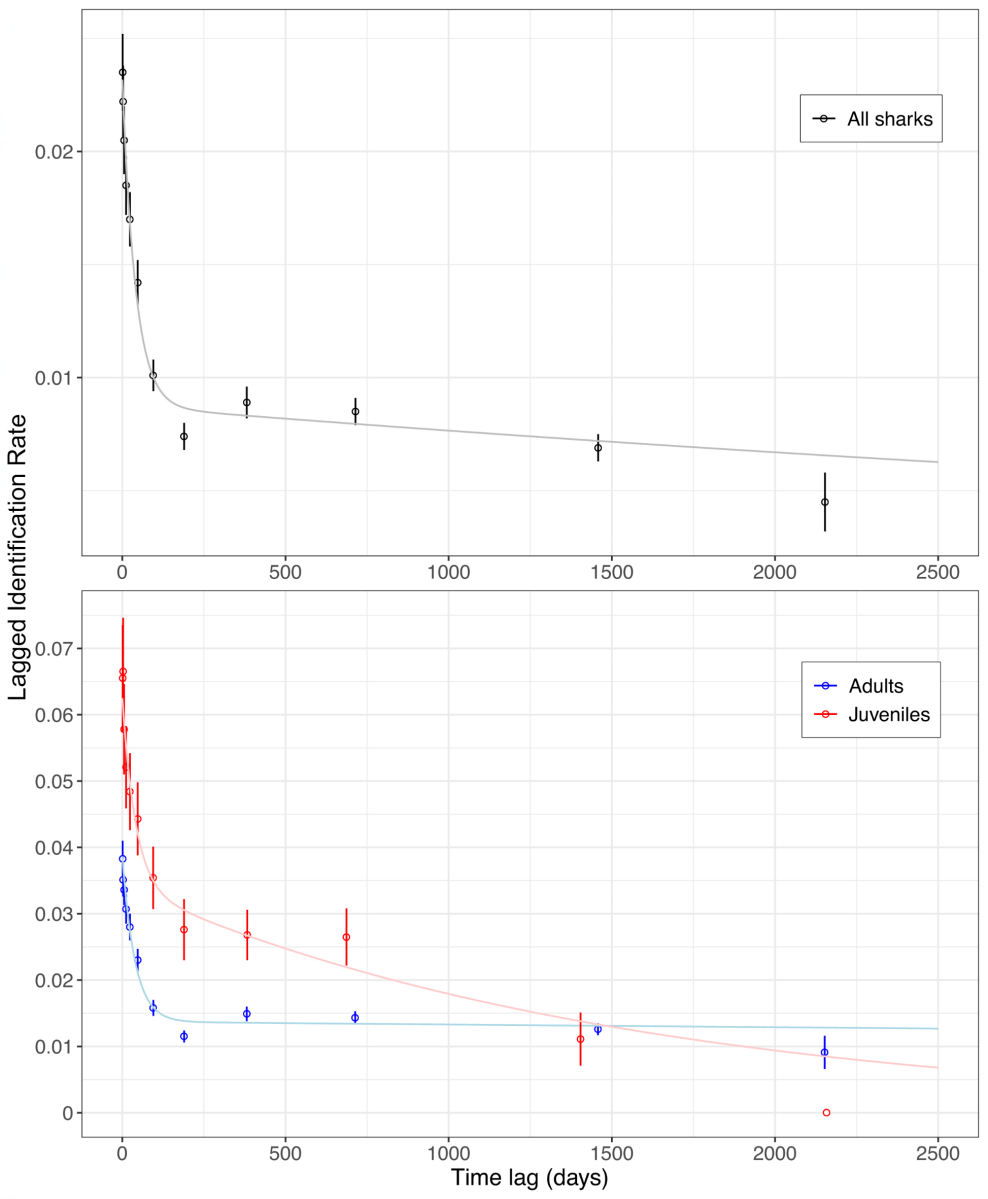
Lagged Identification Rates (LIR, mean ± S.E.) for all tiger sharks (top panel) and for juvenile (red) and adult (blue) female tiger sharks visiting Fuvahmulah. Model H (pale lines) is represented in all cases including emigration, reimmigration and mortality as model presets.

The LIR plot of all sharks showed a rapid decline from day 1 to 190 days (Fig. 5). Afterwards the LIR reached an asymptote at an LIR of 0.007 to 0.009 until 714 days, followed by a further slow decrease to 0.005 until day 2153 never reaching zero. This suggests long-term fidelity to the dive site by at least some individuals. Between 190 and 369 days, the LIR increased, indicating periodicity in the visitation of the dive site by some tiger sharks at half and one-year time periods.

The LIR plot for juvenile females showed high probability of resighting from day 1 to 6 days. LIR remained high until 687 days, or 1.9 years. After 2,158 days the LIR reached zero, implying permanent emigration or mortality (Fig. 5). However, most juveniles have not been identified for periods longer than three years. The LIR plot for adult females displayed a rapid decline from day 1 to 189 days. Afterwards the model curve stabilised at a value of 0.014, never reaching zero. Between 190 and 368 days, the LIR increased significantly and roughly maintained this level until 1457 days, indicating strong periodicity in the visitation of the dive site by some adult female tiger sharks as well as inter-annual site fidelity.

## Discussion

This study provides the first assessment of the population structure, reproductive patterns and residency behaviour of tiger sharks at a hotspot in the central Indian Ocean using non-invasive methods. While adult females showed inter- and intra-annual site fidelity with temporal residency periods, large juveniles showed high residency with shorter periods of absence suggesting that they possibly remain resident in close geographical proximity to the island. Fuvahmulah hosts an unprecedentedly large aggregation of tiger sharks year-round, which appears to play a critical role for the population’s reproductive cycle i.e. as a gestation area for adult females.

### Population structure

The island supports, to our knowledge, the largest documented number of individual tiger sharks encountered in one geographically restricted area with 239 tiger sharks identified in the six-year study period. Few studies about tiger shark aggregations using photo-ID have been published to date. Nakachi (2019) identified 69 individuals from opportunistic photo ID data in Hawai’i over a 16-year study period^49^. Clua et al. (2013) documented the presence of 46 individuals over an eight-day period at a blue whale carcass in New Caledonia^64^. In Tahiti, Bègue et al. identified 55 individuals at a provisioning site over an eight-year study period^65^. The large number of tiger sharks in Fuvahmulah is likely driven by the daily, year-round provisioning activities. While the provisioning activities for tourism started in 2017, fish waste from the local tuna fishery has likely been discarded for generations^48,66^. This low-effort food source probably attracted tiger sharks to the island long before the tourism activities started and altered their distribution in the area. Consequently, we assume that this large aggregation is a consequence of human activity and that sharks within this study likely present a skewed picture of natural tiger shark population dynamics.

The aggregation was dominated by large juvenile and adult females, despite having a sex ratio close to 1:1 *in uterus*^24,30^. This supports size-sex segregation as commonly observed in sharks^20,67,68^ and possibly indicates reproductive needs. The sex-biased habitat use patterns in Hawai’i were found to be driven by the females’ propensity for inshore habitats, whereas males tended to occupy areas farther offshore^37^. Similarly, at Tiger Beach, Bahamas, juvenile and adult females also dominate, with males being almost completely absent except for a few large individuals^47^. Tiger Beach has various similarities to TH in Fuvahmulah: both sites are shallow with warm-water areas adjacent to off-shore, pelagic ecosystems, tiger sharks are provisioned, and pregnant females are observed^47,69^. It has been postulated that the warm waters of Tiger Beach function as a female refuge from male harassment and provide warm temperature gestation grounds^47^. This aligns with the reproductive indications we monitored at TH: adult female sharks stay around Fuvahmulah during their presumed gestation period for extended time periods until they leave for parturition and after an absence period of ca. 2–5 months they return to the dive site. However, male harassment has been witnessed in one photo series during a safety stop in blue waters off TH, where a large male shark swimming significantly faster than the usual cruising speed of tiger sharks was chasing a large, possibly pregnant female (LV, pers. observation). Shortly before contact, both turned away and swam separate ways (supplementary Fig. S2). Fuvahmulah waters do not seem to protect females from male harassment as has been postulated for Tiger Beach^47^. However, intersexual aggression remains a rarity despite the long-term presence of a few adult males at TH (Fig. 2b; authors, pers. observation).

Despite the large number of tiger sharks and the presence of juveniles and adults, small juvenile tiger sharks (< 2.0 m) were not observed surrounding Fuvahmulah. Tiger sharks are known cannibals^70^ and a large diversity of other apex predators including *Sphyrna lewini*, *Carcharhinus albimarginatus* and *C. amblyrhynchos* surrounds the island^71^. Therefore, smaller juveniles probably avoid the area before they reach a size of ca. 2 m TL to evade the elevated predation risk. Such size segregation is common among tiger sharks (e.g.^20,60^).

While visual observations were able to determine sexual maturity in males due to large, calcified claspers, female maturity could not be visually determined. Considering the notable variation in the size at maturity among tiger sharks in the Indian Ocean [females: 3.00 m^21^ to 3.59 m^20^), we recognize that there exists a level of ambiguity regarding the classifications made in this study. Our adoption of > 3.0 m TL as a length-at-maturity is a conservative estimate, more likely to categorise adult individuals ≤ 3.0 m as juveniles rather than the converse.

### Reproductive indications

It was proposed that Indo-Pacific and Atlantic tiger shark populations may have different lengths in reproductive cycles, being biennial in the North Atlantic^24^ and triennial in Hawaiian waters^30^. However, here we show evidence for a biennial reproductive cycle in tiger sharks in the Maldives. Fuvahmulah is located almost on the equator and has a year-round sea surface temperature of 26–30 degrees Celsius (dive log data from dive computers). The continuous presence of warm waters accelerates embryo development and reduces the gestation period^72^. Additionally, the substantial amounts of fish discards provide gestating females a low-effort food source. It is likely that several individuals can temporarily fuel their energetic requirements by scavenging on these discards, a behaviour observed in other large-bodied shark species at similar provisioning sites^73,74^. However, similar studies would be needed to prove this hypothesis. Numerous other potential prey items live in the ecosystems surrounding Fuvahmulah. We filmed how a tiger shark tries to bite a hawksbill turtle *Eretmochelys imbricata* while breathing (Supplementary Fig. S3). Moreover, the island supports a local tuna fishery for yellowfin *Thunnus albacares* and skipjack tuna *Katsuwonus pelamis*. Fishers frequently complain about depredation events^66^. Shark depredation rates can reach up to 26% of all hooked fishes in commercial and recreational fisheries and is energetically highly efficient for sharks^75^. Tunas with massive chunks missing are occasionally observed in the local fishing market, likely due to depredation events by tiger sharks (Supplementary Fig. S4). However, these events have not been quantified for the tuna fishery in Fuvahmulah.

To conclude, we suggest that pregnant females may be using Fuvahmulah waters in part to benefit from the year-round warm waters, and in part to access low-effort food. Similar behaviours have been documented in a variety of other species of pregnant elasmobranchs (e.g.^76–78^). These ideal conditions for reproducing and gestating females may allow this population to reproduce faster than in more challenging environmental conditions with stronger seasonal influences such as Hawaiian waters^79^. However, North Atlantic tiger sharks have similar seasonal influences as Hawaiian tiger sharks and yet exhibit biennial cycles. Unfortunately, data gaps remain throughout the rest of the Indo-Pacific regarding their reproductive cycle. Given the possibility of localised tiger shark populations on smaller geographical scales^34^ and some evidence for biennial reproduction presented here, we recommend avoiding generalisations of reproductive cycle lengths within the Indo-Pacific.

We acknowledge that photographic observations provide limited insight into reproductive biology. Pregnancy was determined based on external appearance, with a consistent abdominal distension over time, which allowed us to quantify the morphological change of sharks before and after presumed parturition. Short-time distended abdomens (e.g. <7 d) likely coincided with the consumption of large amounts of food^64^, and was observed regularly. However, the degree of distension observed in supposedly full-term pregnant females has never been observed in short-time distensions. Combined with the subsequent return to the dive site after a period of absence with considerably altered body conditions, we argue that parturition during this time is extremely likely (Fig. 4).

### Residency

It is common in tiger sharks that large, adult individuals undertake frequent oceanic migrations while displaying site fidelity, whereas juveniles tend to have smaller home ranges (e.g.^37,38,46^). Adult females off Fuvahmulah displayed a high degree of long-term site fidelity. This is evident from the stabilising LIR over time, long absence periods with subsequent returns and the high number of sharks sighted throughout multiple years along the entire study period (Supplementary Fig. S1). In combination with the high resighting rate, this evidence indicates that they are temporal residents with strong inter-annual site fidelity. The long absence periods are likely due to far-ranging migrations either for foraging or for reproductive purposes^37^. Displaying longer residence times and shorter absence periods, juveniles are more likely to stay close to the surrounding waters of Fuvahmulah. We observed several juveniles staying on the periphery of the dive site when adult females were feeding during footage inspection. They tend to approach the site when larger sharks stop feeding (authors, pers. obs.). Since tiger sharks follow strict hierarchical patterns during feeding aggregations^64,80,81^, intraspecific competition makes photographic detection of adult tiger sharks more likely at a feeding site. Hence, a large proportion of juveniles is probably underrepresented in the data set. Therefore, large juveniles (TL > 2.0 m) are potentially resident to the greater area of the waters surrounding Fuvahmulah before reaching maturity and the necessary strength to take on larger migrations^38^. Similarly, large juvenile sharks displayed year-round residency in tropical offshore atoll ecosystems of the Coral Sea^33^. Likewise, small and large juvenile sharks investigated at the Galapagos Marine Reserve spent a remarkable 93% of their time within the reserve^43^.

The inherent limitations of photo-ID at a provisioning site highlights the potential bias towards a fraction of the population i.e. presumably bolder, shallow-dwelling, more aggressive individuals coming in for an easy meal. For instance, several shark species form social networks with non-random associations between individuals (e.g.^82–84^). Despite tiger sharks having been thought to be solitary in nature, evidence from the Bahamas suggest that their associations are in some cases non-random, especially at provisioning sites^85^. Thus, the occurrence of tiger sharks at TH is biased by intraspecific competition, non-random associations, and intraspecific behavioural variations. We hypothesise that TH is dominated by a subset of the population that shows above-average dominance and aggression. Less dominant sharks are likely chased away or avoid close interactions with larger and dominant sharks. At TH, juvenile females generally avoided the feeding area when adult females were present. However, these behaviours are variable and require further investigation. With most photo-ID data from TH (99%), our analyses show the residency to the dive site and not to the waters surrounding Fuvahmulah. It is likely that a large proportion of the population was present in the area but avoided the dive site and thus, remained undetected.

## Conclusions

Fuvahmulah is a bright spot for tiger shark conservation in the Indian Ocean given their protected status within Maldivian waters and being the world’s largest documented aggregation. Likely, female tiger sharks use the area for gestation with strong site fidelity, and thus, the waters off Fuvahmulah serve as a critical habitat for the population. However, the methods applied in this study are limited and provide only initial insights into this aggregation at a provisioning site. It remains unknown where they migrate or where the parturition sites are located. In future studies, we recommend using ultrasonography to confirm the reproductive status of the tiger sharks and to validate our abdominal distention results and assumptions. Satellite telemetry studies on gestating females could indicate their migratory routes when absent^86^, and more interestingly, a novel intrauterine tag (see Sulikowski and Hammerschlag 2023) could indicate exact parturition locations^87^. Furthermore, telemetry methods could reveal their geographic connectivity to other populations and whether Fuvahmulah tiger sharks truly spend most of their time within protected waters of the Maldives shark sanctuary. Nevertheless, the existence of conservation measures in the Maldives is tightly coupled to the economic incentives of shark tourism^10^. Thus, sustainable practices at the provisioning site are of critical importance to provide a net conservation benefit^66,88^. To our knowledge, in the Maldives, there are currently no laws or guidelines regulating provisioned shark dives, and “codes of conduct” are voluntary and dive-centre specific. At other provisioning sites, successful management strategies have been implemented, such as a locally managed MPA in Fiji^89^, guidelines and legislation that regulate SCUBA dives^90^, and policies that manage provisioning activities^91^. To minimize future conflict, we recommend incorporating all stakeholder’s interests into local management plans that support sustainable ecotourism in one of the world’s largest shark sanctuaries.

## Methods

### Study site

The Maldives is a collection of coral atolls that form a chain extending from 7°N to approximately 0.5°S. Fuvahmulah is the biggest single reef island in the Maldives, not being part of a larger atoll and therefore, a true oceanic island along the Chagos-Laccadive Ridge. The Island is located ~ 30 km south of the equator, and is surrounded by a narrow fringing reef with steep slopes reaching to the ocean floor (Fig. 1).

In the harbour area, tiger sharks are now provisioned daily, year-round, with the fishers’ tuna (*Thunnus albacares* and *Katsuwonus pelamis*) discards (i.e. fish heads, guts, bones) to support local dive tourism. This dive site is referred to as ‘Tiger Harbour’ (TH) in this document (Fig. 1). At the dive site ‘Farikede’ (FK), tiger sharks are frequently observed unprovisioned, while exploratory baited dive surveys were conducted in offshore waters (OP) 1.0 to 2.5 nautical miles further south of FK (Fig. 1).

### Photo identification

Footage of tiger sharks was collected from dive guides, recreational divers (citizen scientists) and researchers. Sampling effort was highest from May 2021 until September 2023 due to the presence of a person dedicated to saving collected footage on the island (supplementary Fig. S5). All pictures of sufficient resolution, lighting, and framing of a shark were extracted from the raw footage (supplementary Fig. S6). Individual sharks were identified through a variety of natural markings such as the external pigmentation patterns anterior to the pectoral fins along the counter-shading delineation (Fig. 2a), stripe patterns, and fin shapes^49,64^. A new individual was only added to the catalogue when the footage quality was sufficient to identify at least two different identifiable traits on the left side of the individual (see supplementary Fig. S6 for examples). Additional identifiable traits from both sides were collected further on to enable identification from both sides. The sharks were cross-referenced with a catalogue and each identification was double checked by the principal investigator as well as confirmed by another scientist to minimise human error. Sex was determined from the presence (male) or absence (female) of claspers assessed via sufficient pictures. Maturity in males was assessed through clasper size and calcified appearance^20^. Additional footage of tiger sharks was retrieved during exploratory usage of a remote underwater video station deployed by the Manta Trust and FDS (*n* = 9 encounters).

### Size estimates

Tiger shark sizes were estimated by researchers based on objects of known lengths as a visual reference on the video files and during the dives (i.e. a large chain block, regular dive guides)^92–94^. Estimates for TL were made in 0.5 m increments (e.g. 2.0, 2.5, 3,0 m, etc.). Given that tiger sharks can grow up to 5.5 m TL^23^, we deemed the increments used in our data analysis to be adequate for this study. All estimates were made by the same researcher, who has viewed all footage and conducted > 300 dive surveys in Fuvahmulah. For a subset of sharks, we used laser photogrammetry to control for the accuracy of the estimates. Two adjustable, screw-mounted, green lasers (520 nm, 5 mW) were equipped on a PVC rig 60 cm apart. Videos and pictures were taken on a Panasonic LUMIX GH5 Mk II. Accuracy and distortion of our set-up was assessed following Deakos (2010)^95^. Before each dive, the lasers were calibrated at 3 and 8 m distance using a whiteboard with two dots exactly 60 cm apart. Underwater measurements were made at 3–4 m distance to the sharks for consistency. After each dive, accuracy was reassessed to ensure the laser position had not changed. For each video, at least three frames were extracted, and the average was built for one measurement. Appropriate frames were chosen to minimise parallax error and caudal fin flexing^94–96^. Laser photogrammetry measurements were compared to previous estimations of the same individuals with a paired t-test. TL was obtained from laser photogrammetry PCL measurements using tiger shark morphometrics from La Réunion island^22^. The equation used was:

TL = 1.169*PCL* + 35.697 (r^2^ = 0.98, *n* = 136), from Pirog et al. (2020).

Size differences between sex were compared with the Welch two-sample t-test. Maturity status of female sharks cannot be determined through external appearance. Hence, we used TL to separate mature from immature female individuals and categorised them as adults and juveniles. Based on published size-at-maturity estimates in the Indian Ocean^20–22^, we calculated the mean to designate sharks in this study accordingly. Given a mean of 3.32 m, female sharks that were estimated > 3 m were considered as adults and females estimated ≤ 3 m were considered juveniles. All tiger sharks were assigned their life stage after size estimates from 2022. If the individuals were not present in 2022, their latest size estimate was used.

### Reproductive indications

Adult females were presumed pregnant when an abdominal distension was consistently observed over long time spans of up to five months^69,76,97^. The minimum requirement for a presumed pregnancy was evidence from two separate dives at least two weeks apart consistently showing a similar or increasing degree of abdominal distension. These sharks were scored as ‘pregnant’ in our analysis. Based on an abrupt transition in physical appearance from an abnormally large abdominal distension to a normal appearance or a concave curvature towards the interior of the body, following a period of absence, we inferred that, presumably, parturition had occurred during that time. These sharks were scored as ‘not pregnant’. The presumed pregnancies are referred to as ‘pregnancies’ throughout this manuscript. Based on previous doubts in whale sharks about assigning reproductive status based on visual inspections alone^98^, change in body width throughout presumed pregnancies was documented from photographs^99,100^. Photogrammetric width measures have been shown to successfully detect pregnancies in cetaceans where life history information was known^99,100^. However, this approach has not been applied to sharks yet. Since our data set included photographs of the same sharks throughout their presumed gestation period and subsequent return after parturition, we used this method to quantify the observed morphological changes over time. To develop a dimensionless and scale-invariant metric for comparisons between photographs, we standardised the body widths by a length measure. In this photogrammetry approach, we used the shark’s width from the posterior end of the first dorsal fin vertically down (90° to swimming direction) and the length from the anterior base of the pectoral fin to the anterior base of the pelvic fin to minimise error due to the sharks’ propulsive tail flexing (Supplementary Fig. S7). For this analysis, we used a subset of pregnancies, where the picture quality allowed for the quantification of shark widths by having at least ten sufficient pictures spread over at least six months across presumed pregnancies (supplementary Fig. S7). We calculated the ratios starting one year prior to a shark leaving for apparent parturition and took three measurements after its subsequent return. Sharks that did not fall into the category ‘pregnant’ or ‘not pregnant’, were left as ‘not scored’. To assess if there was a consistent trend in abdominal distension throughout presumed gestation, we applied a linear regression model to the width data until the sharks left.

### Residency and lagged identification rate

Maximum likelihood techniques were used to estimate the parameters of residency models using the software SOCPROG 2.9^101^. These techniques use datasets of individual identifications, where the identifications themselves are used as a measure of effort. Thus, this approach is appropriate for opportunistic data, where sampling periods are distributed neither randomly nor systematically. As such, this method has been successfully applied to various photo identification data sets that are characterised by opportunistic data collection and uneven sampling (e.g.^53,55,102,103^). The models developed by Whitehead (2001) were applied to estimate residency times^55^. These models include various combinations of emigration, immigration, and mortality with preset parameters testing for closed and open population models. The results evaluate the time spent within the study area, the time of absence from the study site after emigration or mortality, and the population size on any given sampling occasion (day). The lagged identification rate (LIR) is the probability that an individual animal is re-sighted at the study site after a certain time lag^55^. LIR plots provide insights into the animal movement and residence behaviour over time and have been applied in this context to whale sharks^53^, manta rays^54^ and cetaceans^55^ amongst others. Goodness of fit of the models was evaluated using the quasi-Akaike information criterion (QAIC) to account for overdispersion of the data^104^. The best-fit model underwent 1000 bootstrap iterations to obtain standard errors of residency parameters and 95% confidence intervals for the calculated LIRs. As juvenile and adult females represent most identifications, models were run for (i) the entire population, (ii) adult females, and (iii) juvenile females. If not stated otherwise, all analyses and visualisations were performed in R (version 4.2.2)^105^.

### Ethics declaration

The study was conducted following the guidelines and under the research permits issued by the Environmental Protection Agency (annually renewable permit: EPA/2021/PA-F01 and EPA/2022/PA-F02) and the Ministry of Fisheries, Marine Resources and Agriculture, Maldives (annually renewable permit: 30-D/PRIV/2021/190). The methods employed were non-invasive in nature, ensuring no harm was caused to the animals involved. All methods used were in accordance with ARRIVE guidelines.

## Electronic supplementary material

Below is the link to the electronic supplementary material.

Supplementary Material 1

## Data Availability

All data is available upon reasonable request to the corresponding author.
