# Supplementary material for: Non-invasive methods characterise the world’s largest tiger shark aggregation in Fuvahmulah, Maldives
Source: Sci Rep. 2024 Sep 23;14:21998. doi: 10.1038/s41598-024-73079-3 (PMC11420367; doi:10.1038/s41598-024-73079-3)
Supplement: Supplementary file 1 — Supplementary Material 1 [file 41598_2024_73079_MOESM1_ESM.pdf]

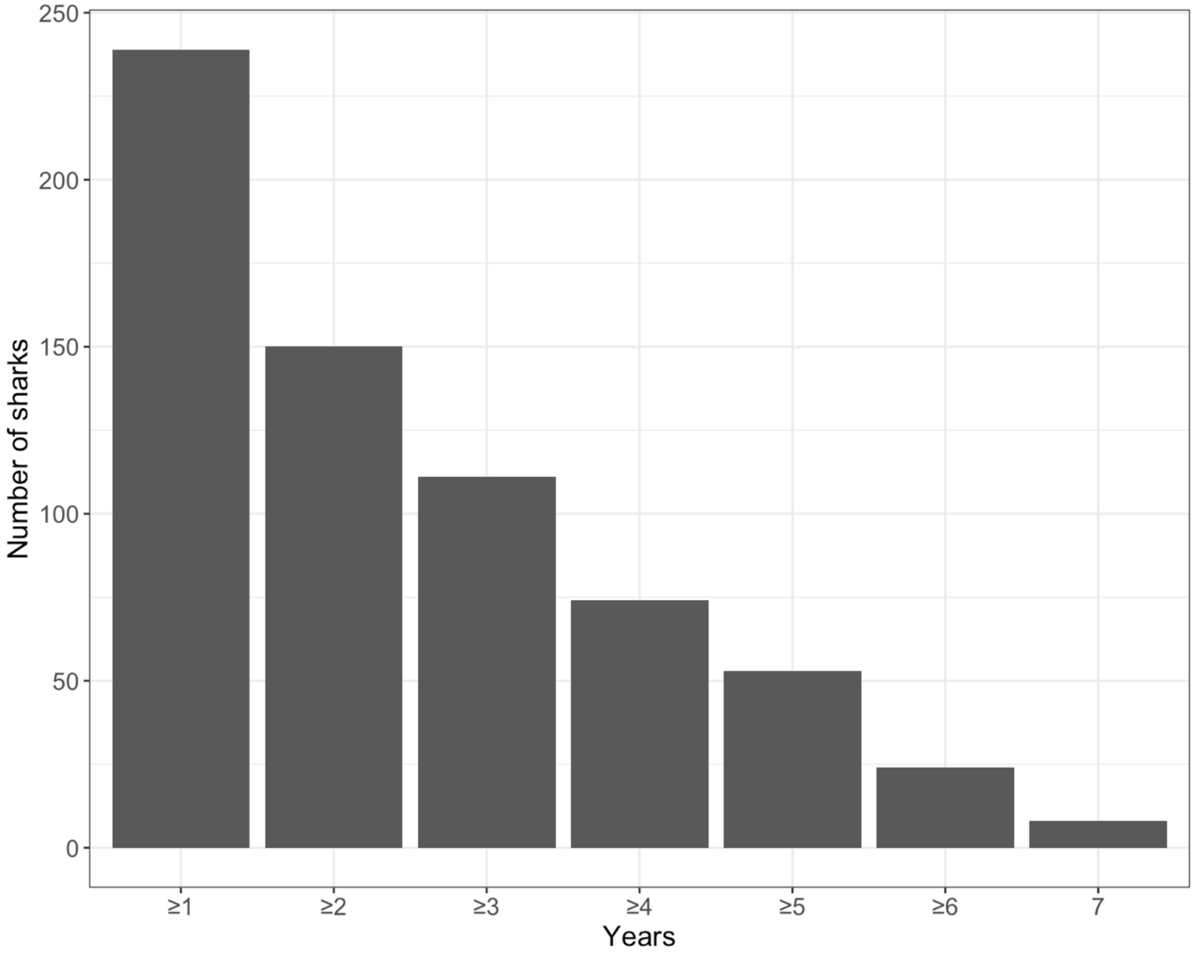

Supplementary Figure S1: The number of sharks identified over multiple years shows the high inter-annual site fidelity of tiger sharks. This information should be viewed with caution because of uneven sampling effort, especially towards year 6 and 7 because of two years (2016, 2020) with low sampling effort (see supplementary Fig. S4).

Shark ID	PCL measured	Scaling to TL	Visual estimate
"F-001"	290.666666666667	375.486333333333	400
"F-005"	282	365.355	350
"F-009"	316.666666666667	405.880333333333	400
"F-011"	290.714285714286	375.542	400
"F-012"	299	385.228	400
"F-018"	288.666666666667	373.148333333333	350
"F-020"	293	378.214	400
"F-021"	271	352.496	350
"F-022"	293	378.214	400
"F-027"	305	392.242	400
"F-033"	278.5	361.2635	350
"F-036"	259	338.468	350
"F-038"	242	318.595	300
"F-045"	280	363.017	350
"F-046"	265.333333333333	345.871666666667	350
"F-054"	284.666666666667	368.472333333333	350
"F-055"	302.833333333333	389.709166666667	400
"F-059"	253.666666666667	332.233333333333	300
"F-061"	265.333333333333	345.871666666667	350
"F-064"	276.5	358.9255	350
"F-065"	287.25	371.49225	350
"F-067"	277.75	360.38675	350
"F-069"	250	327.947	350
"F-071"	243	319.764	300
"F-090"	271	352.496	350
"F-092"	225.333333333333	299.111666666667	300
"F-093"	294.25	379.67525	400
"F-098"	233.666666666667	308.853333333333	300
"F-099"	292.666666666667	377.824333333333	400
"F-103"	286.666666666667	370.810333333333	350
"F-110"	208.333333333333	279.238666666667	250
"F-139"	303.666666666667	390.683333333333	350
"F-113"	303	389.904	400
"F-115"	247	324.44	300
"F-138"	258.75	338.17575	350
"F-140"	300.666666666667	387.176333333333	400
"F-146"	258	337.299	300
"F-147"	254	332.623	350
"F-148"	270.689873417722	352.133462025316	350
"F-151"	250	327.947	350
"F-155"	227	301.06	300
"F-159"	224	297.553	300
"F-162"	277.333333333333	359.899666666667	350
"F-164"	282.5	365.9395	400
"F-167"	202	271.835	250
"F-176"	168.75	232.96575	200
"M-005"	249.75	327.65475	350
"M-006"	260.666666666667	340.416333333333	350
"M-007"	261	340.806	350
"M-009"	265.5	346.0665	350
"M-014"	268	348.989	300
"M-024"	264.5	344.8975	350

Supplementary Table S1: Laser photogrammetry measurements under “PCL measured” were scaled to TL after La R Réunion island tiger shark morphometrics [22]

under and then compared to our visual estimates for quality control of our visual estimates. All numbers are in cm.

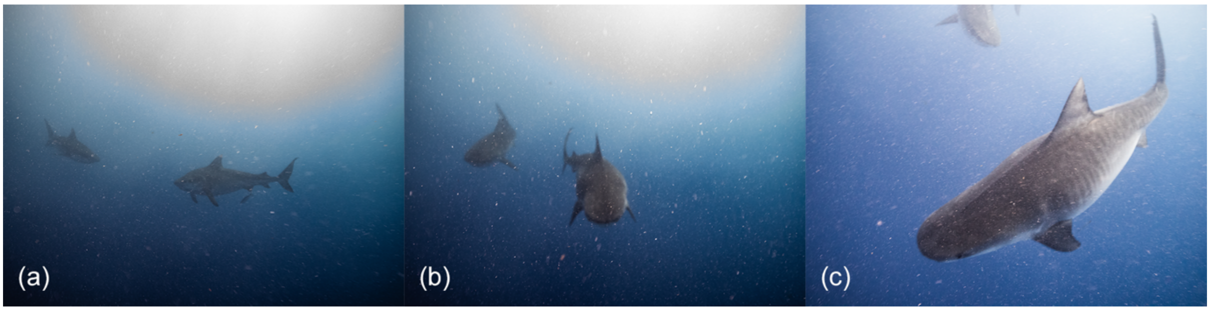

Supplementary Figure S2: Male harassment observed in a photo series. The male shark on the left (M-009, ~3m TL) approached the female (unknown ID, ~3.5m TL) at enormous speed. The female flexed her pectoral fins inwards and turned quickly away from the approaching male (a). The male kept following her (b & c). The female turned in front of the photographer, accelerated once more and both sharks disappeared out of sight (LV, personal observation). The event took place outside of the dive site TH in less than 10m horizontal visibility at approximately 10m depth.

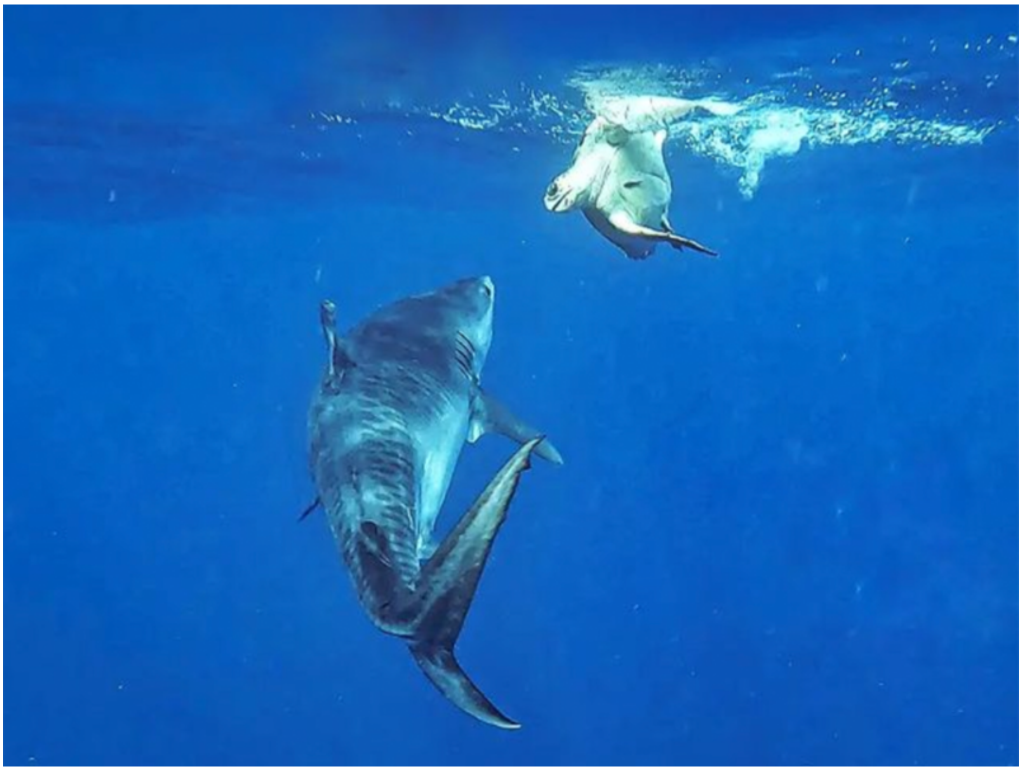

Supplementary Figure S3: Tiger shark predation attempt on a hawksbill turtle *Eretmochelys imbricata*. The attempt was filmed outside of the dive site TH while surfacing from a scuba dive.

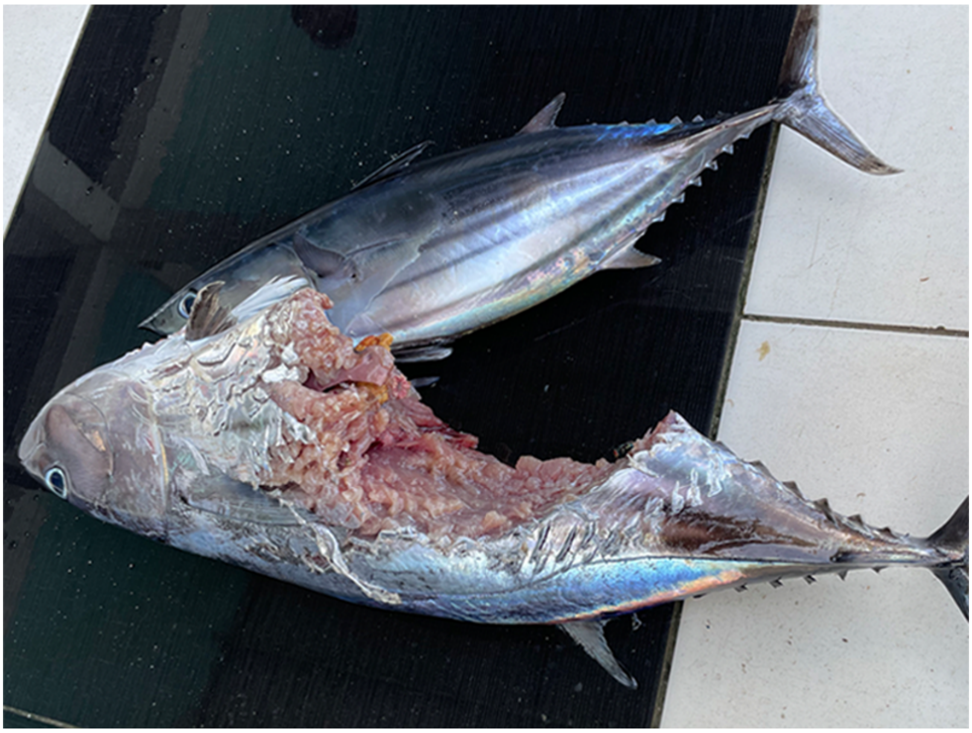

Supplementary Figure S4: Skipjack Tuna missing massive chunks due to depredation. The depredated skipjack tuna *Katsuwonus pelamis* was lilkely bitten by a tiger shark given the size of the chunk missing. It was photographed in the local fish market.

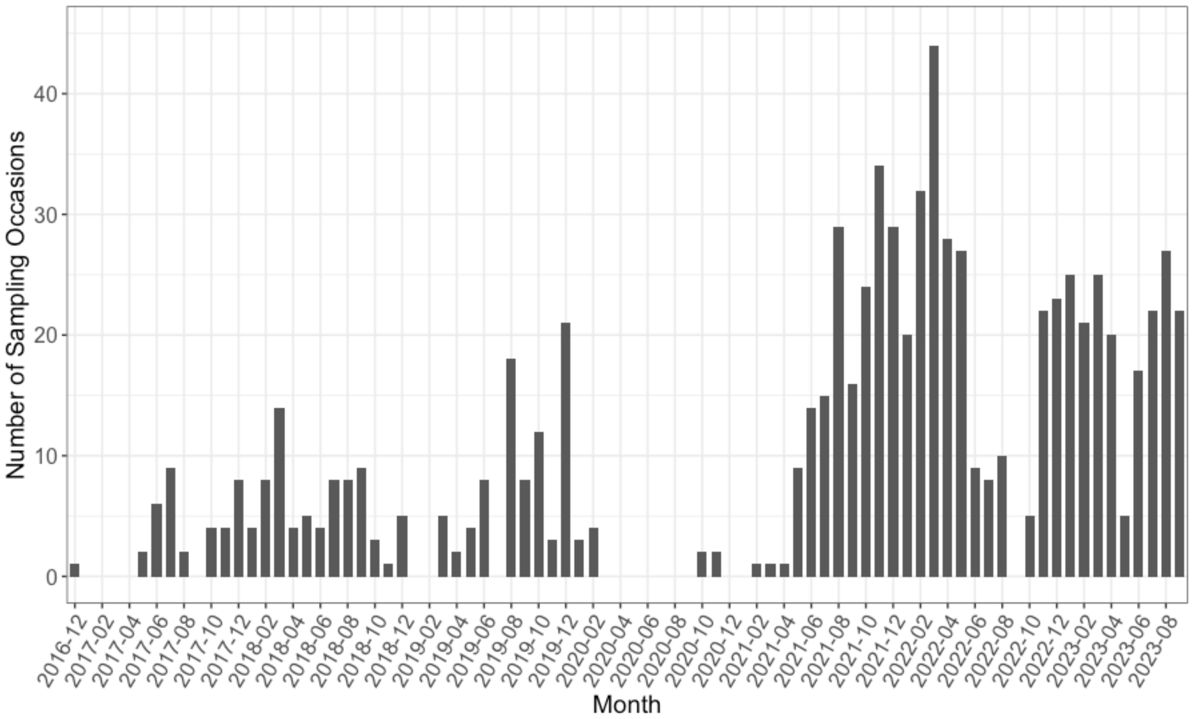

Supplementary Figure S5: Number of sampling occasions per month over the entire study period. Note the high sampling effort from May 2021 until February 2023 due to the presence of a person dedicated to saving collected footage on the island.

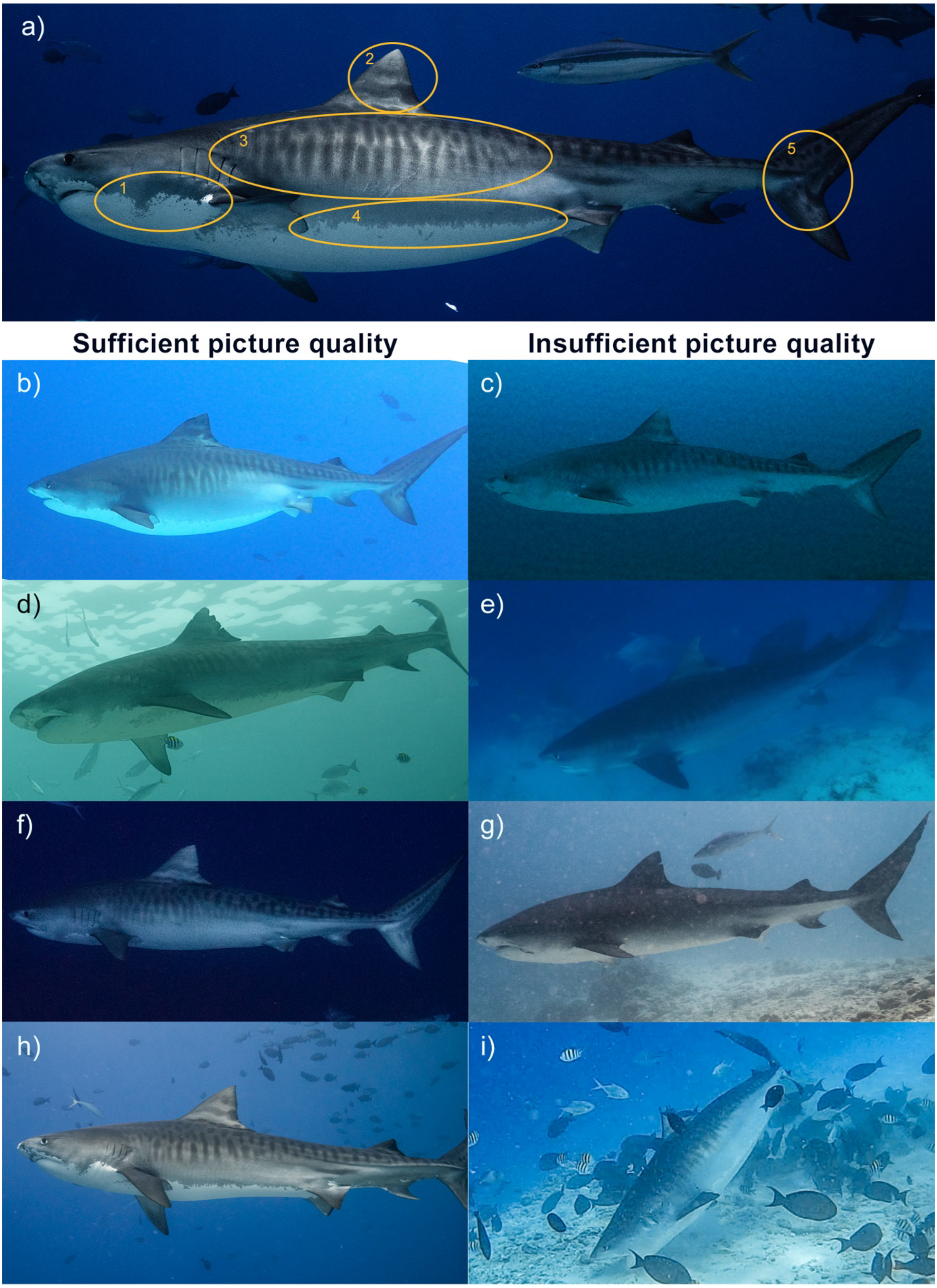

Supplementary Figure S6: Examples for footage quality that allowed for the identification of individuals. a) shows the 5 commonly used identifiable traits for identifying the sharks in this study after Nakachi (2021)[45]. Images b), d), f), and h)

were of sufficient quality by clearly providing at least two identifiable traits. Images c), e), g), i) were insufficient because the image was too dark (c,e), too poor visibility (e, g, i), too low in quality (c), or out of focus (g).

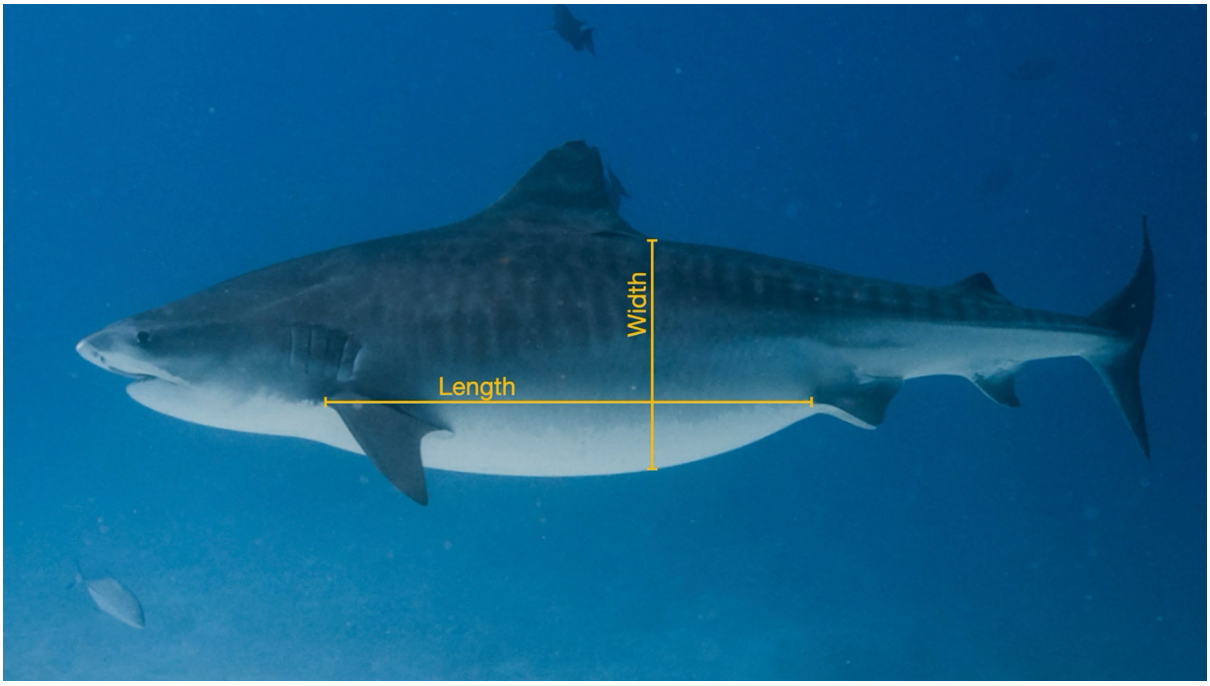

Supplementary Figure S7: Here, we show the width and length measure used to quantify the observed morphological changes. We used the ratio between these two measures to create a dimensionless and scale-invariant standardised width measure.
